# Measurement of the Orbital Soft Tissue Volume in Chinese Adults Based on Three-Dimensional CT Reconstruction

**DOI:** 10.1155/2019/9721085

**Published:** 2019-07-02

**Authors:** Yi Du, Bing-Yao Lu, Jun Chen, Jian-Feng He

**Affiliations:** Department of Ophthalmology, The First Affiliated Hospital of Guangxi Medical University, 6 Shuangyong Road, Nanning 530021, Guangxi, China

## Abstract

Quantitative measurement of the orbital soft tissue volume plays a very important role in the study of orbital diseases. The purpose of this study is to establish a computed tomography- (CT-) based three-dimensional (3D) reconstruction model and measure the orbital soft tissue volume in Chinese adults. We collected data from 103 Chinese adults (52 males and 51 females) who underwent orbital CT. The CT images of these adults were used to reconstruct a 3D model of the orbital bony cavity, orbital fat, extraocular muscle, and intraorbital optic nerve using Mimics software, and their respective volumes were measured. The mean (±SD) orbital bony cavity volume (OV), orbital fat volume (FV), extraocular muscle volume (MV), and intraorbital optic nerve volume (iONV) of the males were 22.2 ± 2.2 cm^3^, 8.9 ± 1.8 cm^3^, 1.9 ± 0.34 cm^3^, and 0.41 ± 0.08 cm^3^, respectively. The mean OV, FV, MV, and iONV of the females were 20.2 ± 1.5 cm^3^, 8.1 ± 1.7 cm^3^, 1.6 ± 0.3 cm^3^, and 0.36 ± 0.074 cm^3^, respectively, which were all significantly lower than those in males (all *p* < 0.05). FV (*r* = 0.370; *p* < 0.001) and MV (*r* = 0.283; *p*=0.007) were significantly correlated with body mass index (BMI), while iONV was not correlated with BMI (*r* = −0.070; *p*=0.480). This study shows that FV, MV, and iONV were higher in males than in females. With increasing BMI, FV and MV both increased, but iONV did not exhibit this trend.

## 1. Introduction

Many ocular diseases can cause volume changes in orbital soft tissues; e.g., thyroid-associated ophthalmopathy can lead to extraocular muscle thickening and increased orbital fat [1], while optic neuritis can cause optic nerve thickening [2]. Therefore, quantitative measurement of the orbital soft tissue volume plays a very important role in the study of orbital diseases.

Presently, the two-dimensional digital tomographic image sequence can be processed by using computers for transformation into a three-dimensional (3D) geometric model with an intuitive three-dimensional effect. Based on computed tomography (CT) or magnetic resonance imaging (MRI), the region of interest can be delineated and transformed into a 3D reconstruction model to calculate the volume of the study object using computer software [3–5]. This method is noninvasive, intuitive, and accurate and a highly feasible method to quantitatively measure tissue volume.

To our knowledge, the orbital soft tissue volume in Caucasians has been well studied, but only a few studies have reported on the normal orbital soft tissue volumes in Asians [6, 7]. However, there are differences in eye parameters between Asians and Caucasians [3–5, 8]. These differences are very unfavorable to study orbital soft tissue diseases in Asian patients with volume changes. This study intended to establish a 3D model of the orbital bony cavity and orbital soft tissues based on CT tomography images and calculate their volumes by computer software. We analyzed the relationship between the orbital soft tissue volumes and physical indicators such as sex, age, height, body weight, and body mass index (BMI). The purpose of this study was to obtain a reference range for the orbital soft tissue volume in Chinese adults.

## 2. Materials and Methods

### 2.1. Ethics Statement

This retrospective study was approved by the Ethical Review Committee of the First Affiliated Hospital of Guangxi Medical University and followed the tenets of the Declaration of Helsinki. Written informed consent was not required because this was a retrospective study.

### 2.2. Subjects

In this retrospective cross-sectional study, data were collected from patients who were admitted to the First Affiliated Hospital of Guangxi Medical University (located in Southern China) and underwent orbital CT from June 1, 2012, to December 1, 2015. The subjects were included in the study if the following conditions were met: (1) they were Chinese patients aged 18 years or older; (2) 16-slice CT scans were performed; and (3) the included patients had no abnormalities on one side.

Patients were excluded if they had the following conditions: (1) an orbital fracture caused by an external force that may have caused an injury that affected the contralateral eye (not necessarily discernible on CT); (2) orbital CT abnormalities were present on the side of interest, the presence of any soft tissue injury, previous eye surgery, high myopia (greater than 600 degrees), or any lesions that might affect the orbital bony cavity or orbital soft tissue (e.g., optic neuritis and strabismus); (3) the presence of thyroid disease, systemic diseases (e.g., hypertension and diabetes), immunologic diseases (e.g., idiopathic thrombocytopenic purpura and systemic lupus erythematosus), and systemic malignancies; and (4) patients with missing images or medical records and those not meeting the standard position requirements (e.g., head tilt or eye position skew).

We collected information on the sex, age, height, and body weight of the patients and calculated the BMI.

### 2.3. CT Scans

All patients were scanned using a 16-slice scanner (Somatom Sensation 16; Siemens Medical Solutions, Erlangen, Germany). A SIEMENS/Sensation 16-slice spiral CT scanner (Siemens Somatom Sensation 16 Computed Tomography) was used. The scanning parameters were as follows: tube voltage, 120 kV; tube current, 88.50 mA; layer thickness, 2.0 mm; slice spacing, 1.0 mm; rack tilt, 0°; and matrix, 512 × 512.

### 2.4. 3D Model Reconstruction

**Figure 1:**
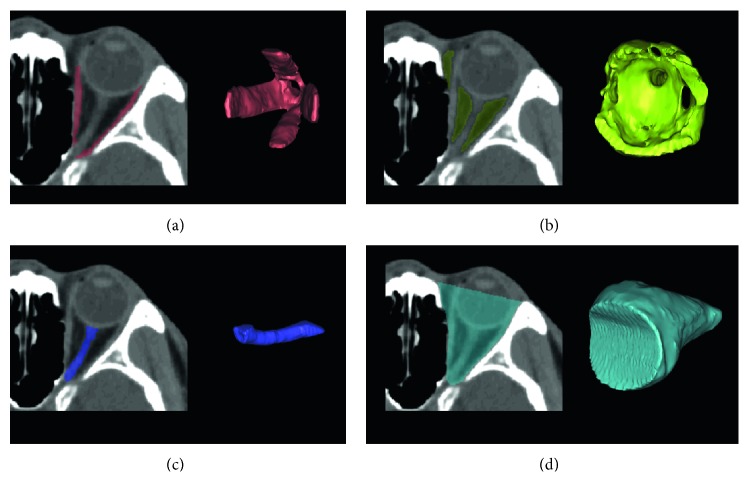
Axial CT slices with the highlighted segmented tissues and the three-dimensional reconstruction of the segmented issues: (a) extraocular muscle, (b) orbit fat, (c) intraorbital optic nerve, (d) orbital bony cavity.

We collected original CT data in DICOM (Digital Imaging and Communications in Medicine) format through the picture archiving and communication systems of the hospital. The medical imaging software Mimics Research (20.0 version; Leuven, Belgium) was used for 3D reconstruction and volume calculation (Figure 1). We used the method proposed by Regensburg et al. [9] to perform 3D reconstruction of the orbital bony cavity, extraocular muscle, orbital fat, and intraorbital optic nerve, and then we calculated the orbital bony cavity volume (OV), extraocular muscle volume (MV), orbital fat volume (FV), and intraorbital optic nerve volume (iONV).

### 2.5. Statistical Analysis

SPSS (SPSS 13.0 for Windows; SPSS Inc., Chicago, IL, USA) was used for the statistical analyses. The comparison of mean values of age, height, body weight, and BMI between men and women was performed using independent sample *t* tests. The mean OV, MV, FV, and iONV values and their ratios (MV/OV, FV/OV, and iONV/OV) were compared using multiple linear regression with and without age adjusted. The correlation between each parameter was analyzed using Pearson correlation analysis. All tests were two-tailed, and the significance level was set at *p* < 0.05.

## 3. Results

One-hundred three patients were enrolled in this study; they were aged 18–81 years, with a mean age (±SD) of 42 ± 15 years. Fifty-two male patients were aged from 18 to 69 years, and 51 female patients were aged from 18 to 81 years. The mean height, body weight, and BMI were 1.61 ± 0.07 m, 56 ± 9.9 kg, and 21 ± 3.3, respectively, for both genders (Table 1).

The results showed that OV, FV, MV, and iONV were all higher in Chinese males than in females (all *p* < 0.05). Regarding the ratios, the MV/OV of males was higher than that of females (*p*=0.017), while the differences in FV/OV (*p*=0.107) and iONV/OV (*p*=0.516) between males and females were not statistically significant (Table 2).

**Figure 2:**
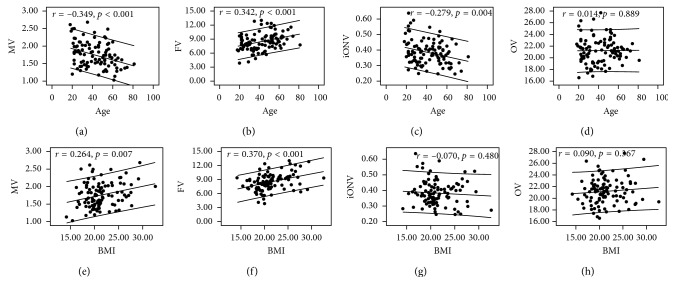
Scatterplots showing correlations between MV, FV, iONV, OV, and age, respectively (a-d), and correlations between MV, FV, iONV, OV, and BMI, respectively (e-h). FV, fat volume; iONV, intraorbital optic nerve volume; MV, muscle volume; OV, orbital bony cavity volume.

With an increase in age, both MV (*r* = −0.349; *p* < 0.001) and iONV (*r* = −0.279; *p*=0.004) decreased, but FV (*r* = 0.342; *p* < 0.001) increased and OV (*r* = 0.014; *p*=0.889) remains unchanged (Figure 2).

OV was positively correlated with height (*r* = 0.516; *p* < 0.001) and body weight (*r* = 0.349; *p* < 0.001) but was unrelated to BMI (*r* = 0.090; *p* < 0.367) (Figure 2). FV was not associated with height (*r* = 0.148; *p* < 0.136) but was positively correlated with body weight (*r* = 0.412; *p* < 0.001) and BMI (*r* = 0.370; *p* < 0.001). MV was positively correlated with height (*r* = 0.491; *p* < 0.001), body weight (*r* = 0.475; *p* < 0.001), and BMI (*r* = 0.264; *p*=0.007). iONV was positively correlated with height (*r* = 0.256; *p*=0.009) but was not associated with body weight (*r* = 0.067; *p*=0.504) and BMI (*r* = -0.070.; *p*=0.480).

## 4. Discussion

In this study, the mean OV of Chinese adult males was 22 ± 2.2 cm^3^, which was significantly higher than that of females (20 ± 1.5 cm^3^). Previous studies have demonstrated that the OV data in Asians, such as Hong Kong people in China (male: 22 ± 1.4 cm^3^; female: 20 ± 2.2 cm^3^) [10] and Koreans (22 ± 1.7 cm^3^ for men and 19 ± 1.8 cm^3^ for women) [11], were similar to ours. However, among Caucasians, the OVs (29 ± 2.4 cm^3^ for males and 25 ± 2.2 cm^3^ for females) [7] were greater than those for the population in this study. Correlation analysis results in this study showed that OV was positively correlated with height and body weight, consistent with the findings of Erkoç et al. [6] and Yoo et al. [12]. We speculate that the smaller OV in our study might be related to the mean body weight and height of Chinese people being smaller than those of Caucasians [13, 14].

Our study showed that with age, OV did not change, FV increased, and MV and iONV decreased. Darcy et al. [15] observed that orbital adipose tissue increased with age, a finding that was similar to ours. In this study, we found that MV decreased with age, consistent with the findings of Yoo et al. [12] and Regensburg et al. [7]. However, Tian et al. [16] reported different results, in which MRI images were used for calculation and indicated that MV was not related to age. The cause may be that the study by Tian et al. included only a few subjects (21 subjects, 42 orbits in either side).

Different from Regensburg et al.'s study [7] of Caucasians, we found that even the ratio of MV to OV (i.e., MV/OV) in males was significantly higher than that of females. Janssen et al. [17] found that the whole body muscle volume in male was significantly higher than that in female. Even with respect to BMI, this difference still exists. The study by Janssen et al. partially supported our findings. Whether there is a difference in the ratio of MV/OV between males and females requires confirmation in further studies.

Many optic nerve diseases, including optic neuritis, optic nerve atrophy, and optic nerve glioma, affect the optic nerve volume [18]. Previously, optic neuritis combined with optic nerve thickening was often misdiagnosed as an optic nerve tumor, and patients underwent unnecessary surgery [19]. Even today, the differential diagnosis of optic neuritis and ischemic optic neuropathy remains a challenge in certain cases, and the thickening of the intraorbital optic nerve is a key point to differentiate the two conditions [2]. Therefore, the range of iONV observed in this study can be used to facilitate the differentiation between these optic neuropathies, especially in the case without MRI.

This study has two limitations. First, the sample size was relatively small, so we did not group the patients by age. Second, in all cases, one side of the orbit is abnormal because the institutional review board did not approve CT scans for normal subjects for research purposes. However, we have attempted to rule out the effect of this abnormal side on the contralateral orbit to be studied.

## 5. Conclusion

In comparison with previous studies, OV, MV, and FV in Chinese tend to be lower than those in Caucasians. This study determined that the MV, FV, and iONV for males were all higher than those for females; even for the ratio of MV/OV, the value for males was still higher than that for females. With an increase in BMI, both MV and FV increased, but iONV did not show this trend.

## Figures and Tables

**Table 1:** Demographic characteristics.

Demographic data	Male (*n* = 52)	Female (*n* = 51)	*p* value
Age, mean ± SD, years	37 ± 12	46 ± 17	**0.002**
Height, mean ± SD, cm	167 ± 5.3	156 ± 4.3	**<0.001**
Weight, mean ± SD, cm	60 ± 8.3	51 ± 9.7	**<0.001**
BMI, mean ± SD	22 ± 3.0	21 ± 3.5	0.586

BMI, body mass index; SD, standard deviation.

**Table 2:** Orbital fat volume, orbital muscle volume, orbital bony cavity volume, intraorbital optic nerve volume, and their ratios in normal Chinese orbits.

	Male (*n* = 52)	Female (*n* = 51)	*p* value	
			Unadjusted	Adjusted for age
OV, mean ± SD, cm^3^	22.2 ± 2.2	20.2 ± 1.5	**<0.001**	**<0.001**
FV, mean ± SD, cm^3^	8.89 ± 1.79	8.09 ± 1.74	**0.024**	**<0.001**
MV, mean ± SD, cm^3^	1.94 ± 0.34	1.57 ± 0.28	**<0.001**	**<0.001**
iONV, mean ± SD, cm^3^	0.405 ± 0.077	0.362 ± 0.074	**0.005**	**0.036**
FV/OV, mean ± SD	0.399 ± 0.063	0.398 ± 0.069	0.937	0.107
MV/OV, mean ± SD	0.0872 ± 0.0131	0.0782 ± 0.0130	**0.001**	**0.017**
iONV/OV, mean ± SD	0.0183 ± 0.0036	0.0180 ± 0.0037	0.702	0.516

FV, fat volume; iONV, intraorbital optic nerve volume; MV, muscle volume; SD, standard deviation; OV, orbital bony cavity volume.

## Data Availability

The data used to support the findings of this study are available from the corresponding author upon request.
